# Chlorogenic acid and its role in biological functions: an up to date

**DOI:** 10.17179/excli2019-1404

**Published:** 2019-06-06

**Authors:** Jae Kwang Kim, Sang Un Park

**Affiliations:** 1 Division of Life Sciences and Convergence Research Center for Insect Vectors, Incheon National University, Incheon 22012, Korea; 2 Department of Crop Science, Chungnam National University, 99 Daehak-ro, Yuseong-gu, Daejeon, 34134, Korea

## ⁯

***Dear Editor,***

Chlorogenic acid (CGA; (IS,3R,4R.5R)-3-{[(2Z)-3-(3,4-dihydroxyphenyl)prop-2-enoyl]oxy}-1,4,5-trihydroxycyclohexanecarboxylic acid) is an ester formed from caffeic acid and quinic acid and works as an intermediate in lignin biosynthesis (Abrankó and Clifford, 2017[1]). CGA, one of the most abundant polyphenol compounds in the human diet, consists of a group of phenolic secondary metabolites isolated from the leaves and fruits of dicotyledonous plants, which is an important component of coffee. CGA has the capacity to manipulate the taste of coffee by modifying astringent, sweet, and sour tastes, which change with the concentration (Tajik et al., 2017[33]).

CGAs are biosynthetically derived from phenylalanine following the phenylpropanoid reaction pathway, which is responsible for the synthesis of several important compounds, like flavonoids, isoflavonoid phytoalexins, coumarins, and lignin (Clifford et al., 2017[6]). There are three possible pathways projected from the p-coumaroyl CoA. Each pathway engages with the same types of enzymatic reactions, such as esterification and hydroxylation (Zhao et al., 2018[49]). These compounds protect plant tissues from damage by oxidative stress, pathogen infection, and wounds. They are also used to mediate animal health (Telles et al., 2017[35]). CGA has a broader range of potential biological properties for health benefits, which might provide non-pharmacological and non-invasive hepatoprotective, antioxidant, anti-diabetic, antimicrobial, anticarcinogenic, anti-inflammatory, and anti-obesity strategies (Maalik et al., 2016[19]; Santana-Gálvez et al., 2017[30]; Tošović et al., 2017[36]; Naveed et al., 2018[24]). Here, we summarize recent literature on the effects of CGAs on different features of health (Table 1(Tab. 1); References in Table 1: Bao et al., 2018[2]; Chen et al., 2017[3]; Chen et al., 2018[4]; Cheng et al., 2019[5]; Ding et al., 2017[7]; Gao et al., 2018[4]; Guo and Li, 2017[9]; Huang et al., 2017[10]; Kaneda et al., 2018[11]; Kato et al., 2018[12]; Kim et al., 2018[13]; Lee and Lee, 2018[14]; Li et al., 2018[15]; Liu et al., 2017[16]; Lou et al., 2016[17]; Ma et al., 2018[18]; Martínez et al., 2017[20]; Mei et al., 2018[21]; Miao et al., 2017[22]; Moghadam et al., 2017[23]; Nguyen et al., 2017[25]; Park et al., 2017[26]; Pereira et al., 2018[27]; Refolo et al., 2018[28]; Sanchez et al., 2017[29]; Siebert et al., 2018[31]; Song et al., 2018[32]; Tan et al., 2016[34]; Vukelić et al., 2018[37]; Wang et al., 2016[38]; Wei et al., 2018[39]; Xue et al., 2017[40]; Yamagata et al., 2018[41]; Yan et al., 2017[42]; Yan et al., 2018[43]; Yang et al., 2017[44]; Ye et al., 2016[45]; Yuan et al., 2017[46]; Yun and Lee, 2017[47]; Zhang et al., 2017[48]; Zhou et al., 2016[50]; Zhu et al., 2018[51]).

EXCLI Journal 2019;18:310-316 – ISSN 1611-2156
Received: May 03, 2019, accepted: June 03, 2019, published: June 06, 2019

| Key findings | Reference |
|---|---|
| A recent study has shown that CGA can effectively act against Al (III)-stimulated cytotoxicity in RAW264.7 cells through the mitogen-activated protein kinase (MAPK)/Akt-mediated caspase pathways. | Cheng et al., 2019 |
| By suppressing the activation of pro-inflammatory and apoptotic signaling pathways, CGA may be very effective against inflammation and attenuating colitis severity. | Vukelić et al., 2018 |
| CGA, which might be used as a potential therapeutic compound for bovine mastitis, had anti-inflammatory activity by inhibiting nuclear factor kappa beta (NF-κB) activation. | Gao et al., 2018 |
| After treatment with CGA, liver injury and insulin resistance could be relieved by suppressing autophagy through the inactivation of the C-Jun N-terminal kinase pathway in a non-alcoholic fatty liver disease (NAFLD) rat model. Based on this finding, CGA could potentially be used for the treatment of NAFLD. | Yan et al., 2018 |
| Crude extract of *Aster koraiensis* acted as a potent antiangiogenic agent. The antiangiogenic activity might be attributable to the bioactive component, CGA. | Kim et al., 2018 |
| CGA, when treated with Regorafenib, improved the anti-cytotoxicity action of Regorafenib in hepatocarcinoma (HCC) cells. This combined treatment as a drug might be a safe and more effective option in HCC therapy. | Refolo et al., 2018 |
| Continuously taking CGA for a period of 6-months improved attention, executive, and memory functions in the elderly who complained of subjective memory loss. | Kato et al., 2018 |
| CGAs improve quality of coffee beverages by decreasing the concentration of 5-O-CGA, while maintain the aroma and taste profile. This might be useful to people with sensitive stomachs. | Siebert et al., 2018 |
| CGA has a significant antidepressant response in sleep-deprived rats. The mechanism of action of CGA may involve the regulation of abnormal pathways, including metabolism of nicotinate and nicotinamide; metabolism of glyoxylate and dicarboxylate; metabolism of glycine, serine, and threonine; and metabolism of arginine and proline. | Ma et al., 2018 |
| CGA stimulates bacterial apoptosis in *E. coli* and the depletion of intracellular ROS (reactive oxygen species), which might regulate the progression of bacterial apoptosis-like death. | Lee and Lee, 2018 |
| Dietary supplementation with CGA enhances the growth performance and reduces the diarrhea incidences of the weaned pigs. It is suggested that CGA improved the antioxidant capacity and enhanced the intestinal digestion and absorption function. | Chen et al., 2018 |
| Semen was effectively stored in 3.2 mg/mL of CGA at 15 °C for up to 24 hr. From this finding, it can be assumed that semen could be stored for longer durations if the CGA concentration increased (6.0 mg/ml or more). | Pereira et al., 2018 |
| CGA, combined with phospholipid complex (PC), improved the post-myocardial infarction inflammatory response in aging hearts, making PC a promising candidate for the clinical development of CGA. | Li et al., 2018 |
| CGA assuaged hepatotoxicity and cholestasis by reducing the uptake and synthesis of bilirubin and bile acids, and finally enhancing the metabolism and efflux of bilirubin and bile acids. | Zhu et al., 2018 |
| By increasing cyclic adenosine monophosphate (cAMP) levels via adenylyl cyclase activation, CGA significantly reduced the carbachol-induced contractions of the mouse urinary bladder. | Kaneda et al., 2018 |
| CGA reduces human lens epithelial cells (hLECs) apoptosis and protects lenses from opacity induced by $H_2O_2$ via the BCL2 (B-cell lymphoma 2) associated X apoptosis regulator (Bax)/ BCL2 apoptosis regulator (Bcl-2) signaling pathway. CGA shows effective defenses against oxidative stress, which could treat a variety of diseases in clinical practice. | Song et al., 2018 |

EXCLI Journal 2019;18:310-316 – ISSN 1611-2156
Received: May 03, 2019, accepted: June 03, 2019, published: June 06, 2019

| Key findings | Reference |
|---|---|
| CGA treatment could inhibit the retinal neoangiogenesis during diabetic retinopathy by nullifying high glucose (HG)-induced hypoxia-inducible factor 1-alpha (HIF-1α)-mediated paracrine vascular endothelial growth factor (VEGF) expression in microglia cells and reducing VEGF-induced angiogenesis in retinal endothelial cells. | Mei et al., 2018 |
| CGA is considered as a therapeutic agent for the treatment of diabetic nephropathy, showing antioxidant and anti-inflammatory effects that are activated via the modulation of the nuclear factor erythroid-derived 2-related factor 2 (Nrf2)/ heme oxygenase-1 (HO-1) and NF-κB pathways. | Bao et al., 2018 |
| CGA prevents acetaminophen (APAP)-induced hepatotoxicity through the Nrf2 antioxidative signaling pathway by blocking the binding of Nrf2 to its deterrence protein, Keap1. Extracellular regulated protein kinase (ERK) 1/2 play a vital role in regulating CGA-induced Nrf2 transcriptional activation. CGA could be utilized as a promising therapeutic agent for the detoxification of APAP-induced hepatotoxicity. | Wei et al., 2018 |
| CGA influences the expression of apoptosis genes related to the oxidative stress and p38 MAP-dependent pathways and the expression of genes encoding stem cell markers. CGA may show polyphenolic anti-cancer effects through the consumption of vegetables and fruits. | Yamagata et al., 2018 |
| By reducing the apoptosis of neuronal cells, CGA mitigates the neuronal damage induced in alcohol exposed neonatal rat. | Guo and Li, 2017 |
| CGA has the potential to relieve liver fibrosis through the miR-21-regulated TGF-β1/Smad7 signaling pathway, which suggests that CGA could act as a new anti-fibrosis agent against liver fibrosis. | Yang et al., 2017 |
| By administering appropriate amounts of myricetin-3-*O*-β-rhamnoside and CGA synergistically, the fibroblastic and remodeling phases of wound repair are accelerated. | Moghadam et al., 2017 |
| CGA controls several mechanisms of action for the development of highly valuable therapeutics for metabolic diseases, such as type 2 diabetes. | Sanchez et al., 2017 |
| CGA shows antifungal activity against phytopathogenic fungi relevant to horticulture and agriculture, suggesting potential biofungicides activity in CGA-enriched wastes and by-products. | Martínez et al., 2017 |
| CGA can modify the gut microbial community structure in the intestines, exhibiting lower intestinal and systemic inflammation, and also enhancing the course of the dextran sulfate sodium-induced colitis, which is linked to a proportional increase in *Akkermansia*. | Zhang et al., 2017 |
| CGA has similar responses to caffeic acid. *In vitro* maturation (IVM) with 50 µM of CGA is particularly useful to the *in vitro* production (IVP) of porcine embryos; 50 µM of CGA prevents oocytes from DNA damage induced by oxidative stress. The addition of CGA to the maturation medium has the potential to enhance the porcine IVP system. | Nguyen et al., 2017 |
| CGA prevents the interleukin (IL)-1β-induced inflammatory response, through the NF-κB signaling pathway. CGA could be a suitable candidate for the treatment of osteoarthritis. | Liu et al., 2017 |
| CGA prevents focal cerebral ischemia reperfusion injury in a rat model by maintaining the inflammatory factor, hypoxia factor, and nerve growth factor. | Miao et al., 2017 |
| CGA prevents the progression of hepatocellular carcinoma via multiple pathways. CGA could be an effective chemopreventive agent for hepatocellular carcinoma. | Yan et al., 2017 |
| CGA retrained benign prostatic hyperplasia in model animals, and this mechanism could protect type Ⅱ 5-alpha reductase activity. | Huang et al., 2017 |
| Consumption of CGA for a period of 5 days significantly enhances fat oxidation during sleep, indicating that beverages containing CGA may be useful to lessen body fat and protect from obesity. Taking CGA also minimizes sleep latency without any adverse effects on sleep quality. | Park et al., 2017 |

| Key findings | Reference |
|---|---|
| CGA can be used as a neuraminidase blocker to prevent influenza A virus both in cellular and animal models, revealing a potential utility for the treatment of the influenza virus infection. | Ding et al., 2017 |
| A self-microemulsifying drug delivery system (SMEDDS) promotes the oral bioavailability of CGA by enhancing its absorption and slowing the metabolism of absorbed CGA by changing its distribution from the liver to the kidney. SMEDDS is an increasingly utilized carrier for the oral delivery of CGA. | Chen et al., 2017 |
| CGA significantly protects mice from Con A-induced hepatitis by reducing the activation of Toll-like receptor (TLR) 4 signaling. This response lowers the expression of adhesion molecules and enhances the infiltration and activation of hepatic leukocytes and the production of pro-inflammatory cytokines. | Yuan et al., 2017 |
| CGA reduces glioma growth by enhancing M1-polarized macrophages and protecting M2 phenotypic macrophages as a potential therapeutic approach. | Xue et al., 2017 |
| CGA promotes potassium efflux, resulting in an apoptotic volume decrease (AVD) and G2/M cell cycle arrest in *Candida albicans*. Thus, potassium efflux via potassium channels improves CGA-induced apoptosis, stimulating several apoptotic processes. | Yun and Lee, 2017 |
| CGA prevents Iso-induced vascular smooth muscle cells (VSMC) damage through the suppression of ROS generation. This finding suggests that CGA could be a novel treatment for vascular diseases. | Wang et al., 2016 |
| CGA prevents both α-naphthylisothiocyanate-induced intrahepatic cholestasis and liver injury. This protective mechanism is modulated through the down-regulation of signal transducer and activator of transcription 3 (STAT3) and NF-κB signaling. | Tan et al., 2016 |
| CGA mitigates the oxidative stress in streptozocin-induced diabetic nephropathy (DN) rats. Its molecular mechanism could prevent the endoplasmic reticulum-stress response in DN. | Ye et al., 2016 |
| CGA is a novel therapeutic agent for preventing inflammatory hyperplasia of the synovium by enhancing synoviocyte apoptosis in patients with rheumatoid arthritis. | Lou et al., 2016 |
| CGA significantly increases hepatic lipid dysregulation in rats by activating fatty acid metabolism enzymes, enhancing AMP-activated protein kinase activation, and modulating levels of hepatic fatty acids. | Zhou et al., 2016 |

## *Conflict of interest*

The authors declare no conflict of interest.

## Acknowledgements

This work was supported by a grant from the Next-Generation BioGreen 21 Program (SSAC, Project #. PJ013328)" Rural Development Administration, Republic of Korea.

## Conflict of interest

The authors declare no conflict of interest.

## Figures and Tables

**Table 1:**
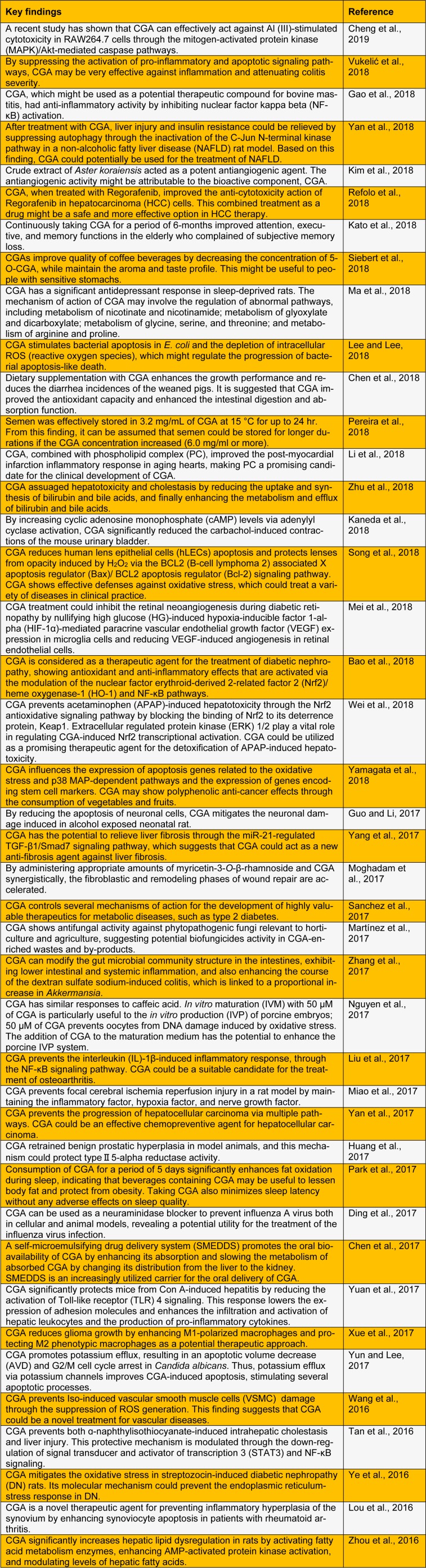
Recent studies of the biological and pharmacological activities of chlorogenic acid
